# Supplementary material for: The Investigation of the Influence of a Cu2O Buffer Layer on Hole Transport Layers in MAPbI3-Based Perovskite Solar Cells
Source: Materials (Basel). 2022 Nov 17;15(22):8142. doi: 10.3390/ma15228142 (PMC9699280; doi:10.3390/ma15228142)
Supplement: Supplementary file 1 [file materials-15-08142-s001.zip › materials-1987502-supplementary.pdf]

materials

The Investigation of the Influence of a Cu_2O Buffer Layer on Hole Transport Layers in $MAPbI_3$-Based Perovskite Solar Cells

Chunxiang Lin [1], Guilin Liu [1]*, Xi Xi [1], Lan Wang [2], Qiqi Wang [1], Qiyan Sun [1], Mingxi Li [1], Bingjie Zhu [3], David Perez de Lara [4] and Huachao Zai [5]

1. School of Science, Jiangnan University, Wuxi 214122, China
2. School of Internet of Things, Jiangnan University, Wuxi 214122, China
3. Wuxi Institution of Supervision & Testing on Product Quality, Wuxi 214101, China
4. Zhejiang Beyondsun Green Energy Technology CO., LTD., Huzhou 313200, China
5. School of Materials Science and Technology, China University of Geosciences (Beijing), Beijing 100083, China

* Correspondence: guilinliu@jiangnan.edu.cn

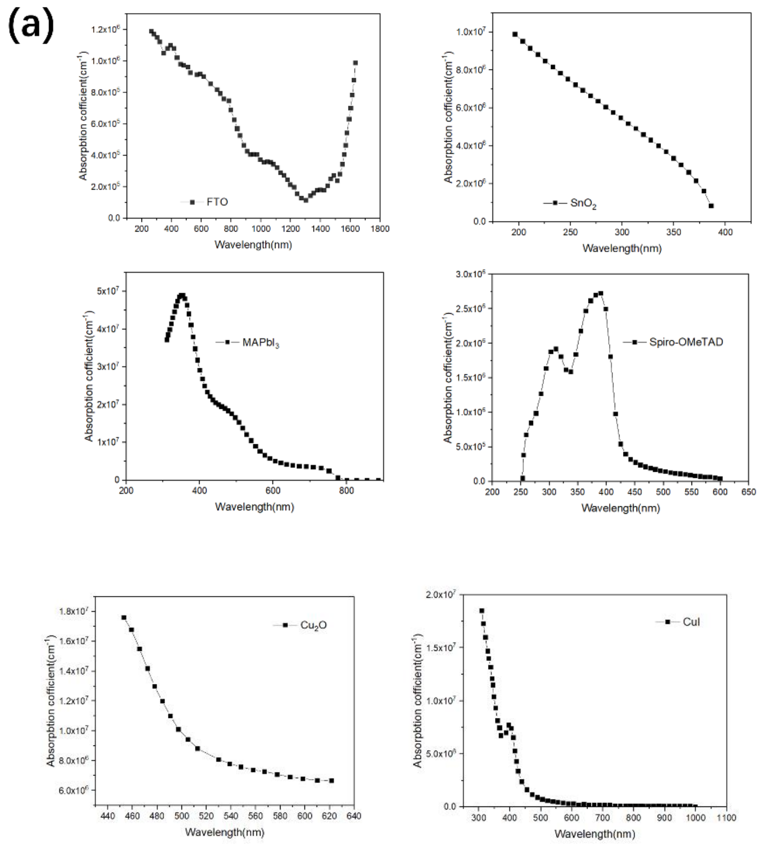

(b)

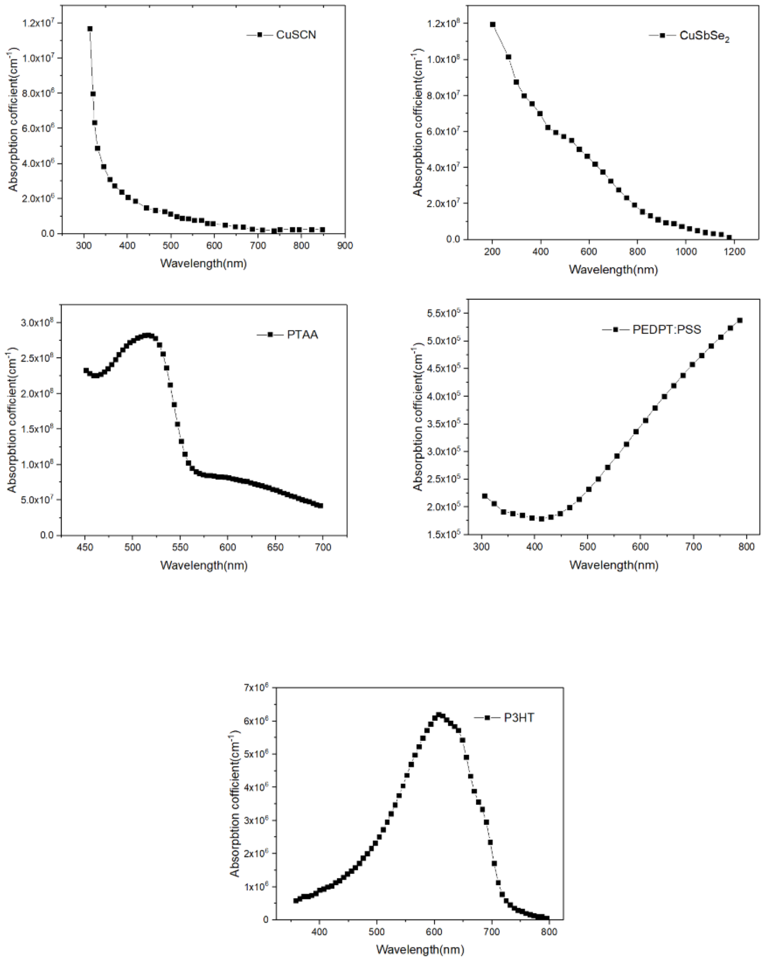

Figure S1. Absorption coefficient curves of FTO, SnO_2, $MAPI_3$, Cu_2O and HTLs. The data used to plot these curves are obtained from results reported (FTO [56], SnO_2[57], MAPbI3[58], Spiro-OMeTAD[59], Cu2O[60], CuI[61], CuSCN[62], CuSbSe2[47], PTAA[63], PEDOT:PSS[64], P3HT[65]).

Table S1. Parameters of interface layer.

Interface	Defect type	Capture cross section electrons/holes (cm^2)	Energetic distribution	Reference for defect energy level Et	Energy with respect to Reference (eV)	Total density (integrated over all energies) ($1/cm^2$)
ETM/$MAPbI_3$	Acceptor	1×10^{-17}<br>1×10^{-18}	Single	above the highest E_v	0.32	1×10^9
$MAPbI_3$/HTM	Acceptor	1×10^{-18}<br>1×10^{-19}	Single	above the highest E_v	0.07	1×10^9
Reference				[19]		

Table S2. Parameters of back and front contacts.

Metal contacts properties	Back contact	Front contact
Surface recombination velocity of electrons(cm/s)	1×10^5	1×10^7
Surface recombination velocity of holes(cm/s)	1×10^7	1×10^5
Metal work function(eV)	Adjustable	4.4

Table S3. Work Function of Back Metal electrode.

Back Metal contact	Cr	Cu	Ag	Au	Ni	Pt
Work function(eV)	4.5	4.65	4.7	5.1	5.15	5.65
Reference	[66][67]					

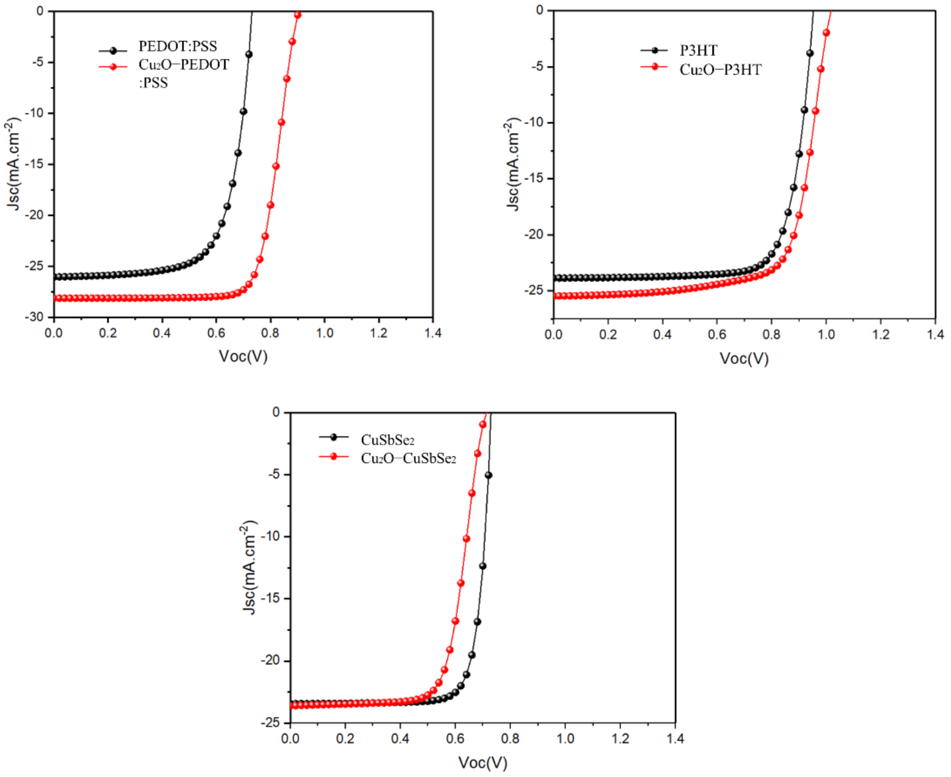

Figure S2. Current density- voltage (J−V) characteristic curves of both ideal device structures (with and without Cu_2O buffer layer) with PEDOT: PSS, P3HT, and $CuSbSe_2$ HTLs.
